# Paromomycin Reduces *Vairimorpha (Nosema)* *ceranae* Infection in Honey Bees but Perturbs Microbiome Levels and Midgut Cell Function

**DOI:** 10.3390/microorganisms10061107

**Published:** 2022-05-27

**Authors:** Rachel M. Cho, Helen V. Kogan, Annabelle B. Elikan, Jonathan W. Snow

**Affiliations:** Department of Biology, Barnard College, Columbia University, New York, NY 10027, USA; rmc2201@barnard.edu (R.M.C.); hvk2105@columbia.edu (H.V.K.); abe2120@barnard.edu (A.B.E.)

**Keywords:** *Vairimorpha*, *Nosema*, microsporidia, honey bee, infection, paromomycin, cell stress

## Abstract

Paromomycin is a naturally occurring aminoglycoside antibiotic that has effects on both prokaryotic and eukaryotic microbes. However, previous reports have indicated that it has little effect on microsporidia, including *Vairimorpha (Nosema) ceranae*, in cell culture models. *V. ceranae* is one of a number of microsporidia species that cause disease in honey bees and substantial efforts to find new treatment strategies for bees that are infected with these pathogens are ongoing. When testing compounds for potential activity against *V. ceranae* in whole organisms, we found that paromomycin reduces the infection intensity of this parasite. Critically, the necessary doses of paromomycin have high activity against the bacteria of the honey bee microbiome and cause evident stress in bees. Microsporidia have been shown to lack an essential binding site on the ribosome that is known to allow for maximal inhibition by paromomycin. Thus, it is possible that paromomycin impacts parasite levels through non-cell autonomous effects on microsporidia infection levels via effects on the microbiome or midgut cellular function. As paromomycin treatment could cause widespread honey bee health issues in agricultural settings, it does not represent an appropriate anti-microsporidia agent for use in the field.

## 1. Introduction

Microsporidia are obligate intracellular parasites that cause infections in a wide range of hosts, but they have been relatively understudied compared to microbial pathogens representing other taxonomic groups, such as bacteria [1]. The *Vairimorpha* (formerly *Nosema* [2]) species, *ceranae* and *apis,* are microsporidian parasites that are pathogenic to honey bees and infection by these species has been implicated as a key factor in honey bee losses [3,4,5]. Environmental spores that are shed from infected bees are consumed by uninfected bees, upon which the spores first inject sporoplasms into the cells of the host’s midgut. The sporoplasms then develop into meronts that begin to rapidly proliferate before maturing into sporoblasts. The latter produce large numbers of primary spores and, ultimately, new infective environmental spores, which are then released from the infected cell to begin the cycle anew [6]. Midgut infection by *V. ceranae* causes disruptions in tissue structure and function, leading to energetic stress [3,4,5]. At the organismal level, infection is associated with reduced survival rates and a number of physiological and behavioral changes that reduce individual contribution to the colony [3,4,5]. *V. ceranae* infection has traditionally been treated with the drug fumagillin in the United States, but its use is prohibited in Europe (reviewed in [7]) and its effectiveness and durability in controlling *V. ceranae* at the colony level are in question [8]. Additionally, fumagillin may impact host cell function at high doses, *V. ceranae* may be able to evade suppression in some circumstance [9], and the future availability of fumagillin is also uncertain. Many promising alternative strategies for the mitigation of *V. ceranae* infection are now being pursued (see [10] and references therein).

Paromomycin is a naturally occurring aminoglycoside antibiotic, produced by *Streptomyces rimosus,* that affects both prokaryotic and eukaryotic microbes by binding to the A-site of the small subunit of the ribosome [11]. Previous studies have found variable effects of paromomycin on microsporidia, which are predicted to be resistant to paromomycin based on ribosome RNA sequences [12]. An early study showed a small effect of paromomycin on the prevalence of *V*. *apis* infection in caged honey bees [13]. However, subsequent studies on *Encephalitozoon* sp. have shown no impact of paromomycin in a cell culture-based system of *E. cuniculi* infection [14], nor in a patient-based study of *E. bieneusi* infection [15]. More recently, a cell culture-based system of *V. ceranae* infection also revealed no impact of paromomycin on infection levels [16]. However, this study was performed using a lepidopteran cell line from an entirely different insect order than the natural bee hosts of this parasite, in which only a single round of infection is achievable [16,17]. Based on the initial promising results on *V. apis* and the caveats associated with the cell culture-based system utilized for studies on *V. ceranae*, we felt further study was warranted. We therefore investigated the impact of paromomycin on *V. ceranae* infection in caging infection experiments with honey bees.

## 2. Materials and Methods

### 2.1. Honey Bee Colonies and Caging Experiments

The honey bee experiments were performed as before [10,18]. Source colonies for the bees were outbred colonies in New York, New York, consisting of a typical mix of the *Apis mellifera* subspecies that is found in North America. The bees were collected at different times during the months of April–October from colonies that were visually inspected for symptoms of common bacterial, fungal, and viral diseases. For the caging experiments, newly emerged bees were collected after hatching from a capped brood frame overnight in an incubator at 35 °C in the presence of PseudoQueen (Contech, Victoria, BC, Canada) as the source of queen mandibular pheromone (QMP), which partially mimics queen presence and reduces stress when workers are caged alone. Approximately 30 newly emerged bees were placed in each 12.2 cm × 8.6 cm × 21.3 cm acrylic cage with a sliding door, machined at Carelton Labs, Columbia University. The newly emerged caged bees were fed a 33% sucrose solution and supplied with a ~5 g pollen substitute patty (1:1 mix of BeePro and sucrose solution). Approximately 4 foragers from the same source colony (marked with a spot of paint (Testors, Vernon Hills, IL, USA)) were added to each cage to facilitate the growth of the microbiome. When older bees were used, ~20 bees collected from the landing board of a colony were placed in cages as above and fed a sucrose solution.

### 2.2. Isolation and Quantification of V. ceranae Spores

*V. ceranae* spores were obtained from infected individuals for use in these infection studies [10]. In addition, an isolate was obtained from this colony and serially passaged through the bees, as performed previously [19]. Spores from these bees were used in some experiments. To isolate spores, the midguts of infected bees were individually crushed in 0.5 mL of H_2_O and the spore number was assessed by light microscopy. Midguts were washed 3 times with water by repeated centrifugation and resuspended in 33% sucrose solution at a concentration of 1 × 10^6^ spores per mL for landing board bees or 5 × 10^6^ spores per mL for newly emerged bees.

### 2.3. Infections and Chemical Treatments

For newly emerged bees, *V. ceranae* spores (5 × 10^6^ per mL) were immediately fed to the bees ad libitum in a sucrose solution [20] for 48 h. For experiments with landing board bees, the caged bees were allowed to consume food containing spores (1 × 10^6^ per mL) ad libitum for 24 h before the food was replaced with sucrose solution alone. At three days post-infection, honey bees in individual cages were fed sucrose solution alone or sucrose solution containing paromomycin (at doses from 0.25 to 1 mg/mL). After 4 days of drug feeding, the honey bee midguts were dissected and crushed in 0.5 mL of water, then the number of mature spores was counted by light microscopy [21], as previously described [10,18]. In parallel, qPCR was used on harvested midgut tissue (pooled from bees in the specific group) to determine the relative amount of *V. ceranae* genome equivalents versus host genome equivalents. Bees in the uninfected group always received sucrose solution containing the midgut of an uninfected bee, which was processed in the same way as the midguts containing spores. All experiments were performed 2–4 times.

For survival experiments and gene expression analysis on uninfected bees, newly emerged bees were caged and fed as above. For survival, bees were switched to sucrose solution alone or sucrose solution containing paromomycin at the indicated dose for 10 days, starting 4 days post-eclosion, while survival was assessed. For biomarker gene expression, bees were switched to sucrose solution alone or sucrose solution containing paromomycin (1 mg/mL) for 4 days, starting 6 days post-eclosion, prior to dissection and gene expression analysis.

### 2.4. DNA Extraction and qPCR

DNA extraction was performed using a modified “smash and grab” DNA miniprep protocol, as described previously [10]. The resulting DNA was used as a template for qPCR to determine the levels of infection of *Vairimorpha* sp. using primers for the *V. apis 16S* gene and the *V. ceranae β-actin* relative to the honey bee *ATP5a* gene [18,22]. For the qPCR reactions, PowerUp SYBR Green Master Mix (Applied Biosystems, Foster City, CA, USA) was used in accordance with the manufacturer’s instructions in a LightCycler 480 thermal cycler (Roche, Branchburg, NJ). The PCR conditions were as follows: 94 °C for 2 min, followed by 94 °C for 15 s, 60 °C for 30 s, and 72 °C for 60 s for 40 cycles. These steps were followed by a 10 min extension step at 72 °C. The difference between the threshold cycle (Ct) number for honey bee *ATP5a* and that of the *Vairimorpha* sp. of interest was then used to calculate the relative level infection using the 2^(−^^ΔCT)^ method [23]. A sample was considered negative for a specific *Vairimorpha* sp. when it did not amplify any product by 35 cycles and 0 was entered as the value in these cases. For examining the levels of total bacteria and specific bacterial species in the digestive tract microbiome (*Gilliamella apicola*, *Frischella perrara*, *Snodgrassella alvi*, *Bartonella apis*, *Bifidobacterium asteroids*, *Lactobacillus* Firm-4, and *Lactobacillus* Firm-5), a similar assay was performed using universal 16S rRNA primers and species-specific 16S rRNA primers from [24], in conjunction with the honey bee *ATP5a* gene.

### 2.5. RNA Isolation, Reverse Transcription, and Quantitative PCR for Gene Expression Analysis

RNA was prepared from the midgut tissue of the bees, as previously described [25]. Midgut tissue was manually crushed with a disposable pestle in Trizol Reagent (Invitrogen, San Diego, CA, USA) and RNA was then extracted as per the manufacturer’s instructions. RNA was then DNaseI-treated by RQ1 RNase-Free DNase (Promega, Madison, WI, Canada) and cDNA was synthesized using approximately 1 μg of RNA and the High-Capacity cDNA Reverse Transcription Kit with RNase Inhibitor (Applied Biosystems, Foster City, CA, USA). For the quantitative PCR (qPCR) reactions to determine the expression levels of the gene of interest, 1 μL of cDNA was used as a template, in conjunction with PowerUp SYBR Green Master Mix (Applied Biosystems, Foster City, CA, USA) and appropriate primers. Reactions were run in a LightCycler 480 thermal cycler (Basel, Switzerland) or Bio-Rad CFX Opus (Bio-Rad, Hercules, CA, USA) using the PCR conditions stated above. The primer sequences targeting the transcripts of the gene of interest were from [25]. The difference between the threshold cycle number for *β-actin* and that of the gene of interest was used to calculate the level of that gene relative to *β-actin* using the typical 2^(−^^ΔCT)^ method [23]. All qPCR data represent the expression values from individual bees (sample sizes found in figure legends) and is displayed as the mean ± SEM.

### 2.6. Statistical Analysis

Data are presented as mean ± SEM. For two groups, data were compared using unpaired *t*-tests with Welch’s correction when the values fit normal distributions and Mann–Whitney U nonparametric tests when they did not fit normal distributions. Normality was assessed using Shapiro–Wilk tests. When more than two groups were compared, data were compared using one-way ANOVA with Tukey’s multiple comparison test when values fit normal distributions and Kruskall–Wallis tests when they did not. For survival analysis, treated versus untreated groups were compared using the Mantel–Cox test.

## 3. Results

### 3.1. Paromomycin Reduces vs. Ceranae Infection Intensity in Honey Bees

**Figure 1:**
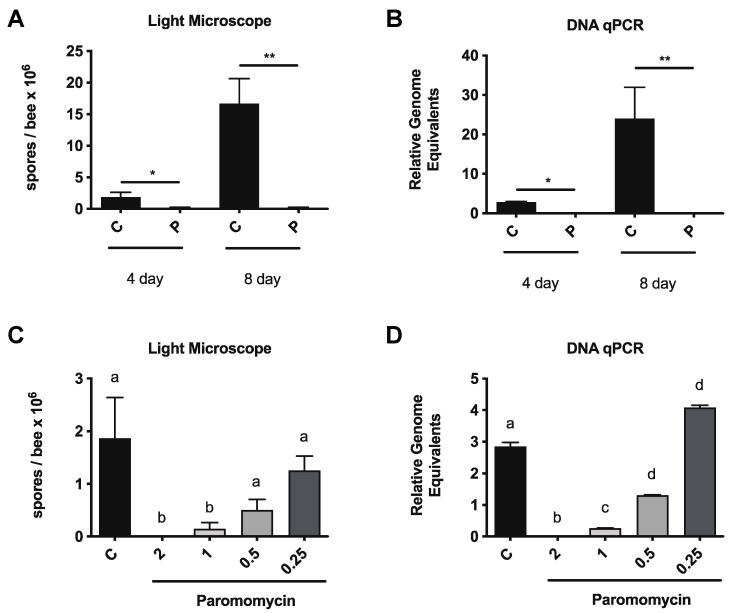
**Paromomycin treatment reduces *V. ceranae* infection levels in newly emerged bees and colony bees**. *V. ceranae* levels in the midguts of infected newly emerged bees fed with a sucrose solution with (*n* = 14) or without (*n* = 14) paromomycin for 4 or 8 days, as determined by spore count using light microscopy (**A**) or qPCR (**B**). *V. ceranae* levels in the midguts of infected newly emerged bees fed with a sucrose solution containing various doses of paromomycin (*n* = 15, 2 mg/mL; *n* = 14, 1 mg/mL; *n* = 14, 0.5 mg/mL; *n* = 12, 0.25 mg/mL) for 4 days, as determined by spore count using light microscopy (**C**) or qPCR (**D**): Statistical significance is noted as * for *p* < 0.05 and ** for *p* < 0.01 or a ≠ b ≠ c ≠ d; *p* < 0.05.

To obtain age-matched bees and allow for longer treatment periods, we used newly emerged bees and tested the effects of paromomycin on *V. ceranae* infection intensity. On day 2 post-eclosion, *V. ceranae* spores (5 × 10^6^ mL) were fed to bees ad libitum in a sucrose solution [20] for 48 h. At 3 days post-infection, honey bees in individual cages were fed a sucrose solution containing paromomycin at the indicated dose or sucrose solution alone. Honey bee midguts were dissected after 4 or 8 days of drug feeding and infection levels were assessed by spore counting and qPCR. We observed reductions in infection levels, as assessed by spore counting, and relative genome equivalents after paromomycin treatment (Figure 1A,B).

We then tested the ability of paromomycin to reduce *V. ceranae* infection in bees that were taken directly from the colony. After experimentally infecting the bees from the landing board, which were likely foragers, we fed the infected bees with a sucrose solution or sucrose solution containing 1 mg/mL of paromomycin for 4 days, starting 3 days post-infection. On Day 8 post-infection, we measured the spore levels using light microscopy and the amounts of *V. ceranae β-actin* gene relative to the honey bee *ATP5a* gene by qPCR to determine the effects of proteasome inhibition on the *V. ceranae* infection intensity. We found that feeding infected bees paromomycin for 96 h resulted in a dramatic reduction in infection intensity, according to both measures, compared to untreated bees (Figure S1A,B).

We then performed a dose response experiment, in which we infected newly emerged bees as above and fed them paromomycin at 0.25, 0.5, 1, and 2 mg/mL, starting on day 3 post-infection. After 4 days of drug feeding, the honey bee midguts were dissected and infection levels were assessed by spore counting and qPCR. We observed reductions in infection levels, by spore counting, and relative genome equivalents for paromomycin in a dose-dependent manner (Figure 1C,D).

### 3.2. Paromomycin Impacts on Microbiome Levels

**Figure 2:**
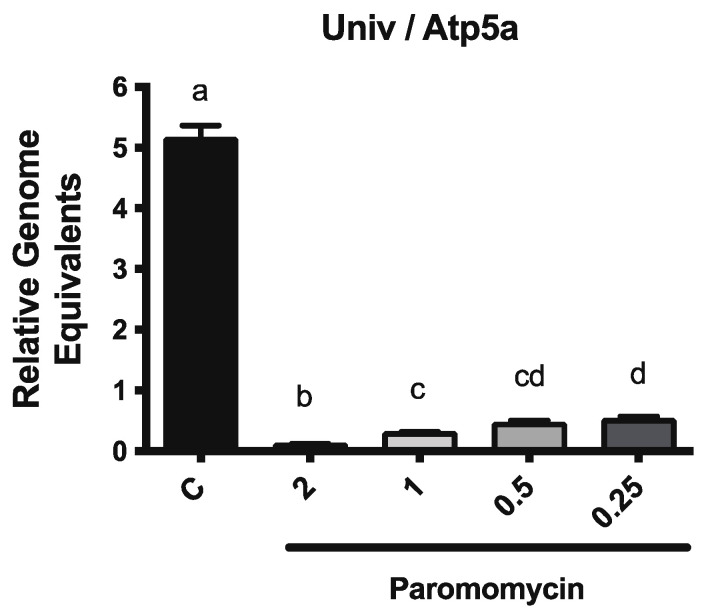
**Paromomycin impacts honey bee microbiota**. Levels of total bacteria, as determined by qPCR, in the midguts of infected newly emerged bees fed with a sucrose solution containing various doses of paromomycin for 4 days: a ≠ b ≠ c ≠ d; *p* < 0.05.

To determine whether paromomycin treatment resulted in alterations to the microbiome, we used qPCR to measure the levels of the core bacterial species that are known to be part of the honey bee digestive tract microbiota. We used primer sets to amplify the 16S rRNA region of all bacteria, as well as sets that amplified the species-specific 16S rRNA region of seven bacterial species, including *Gilliamella apicola*, *Frischella perrara*, *Snodgrassella alvi*, *Bartonella apis*, *Bifidobacterium asteroids*, *Lactobacillus* Firm-4, and *Lactobacillus* Firm-5. We found that paromomycin dramatically reduced the total bacteria levels in the midgut at all tested doses, as expected (Figure 2). We also observed changes in the relative amounts of individual bacterial groups (Figure S2).

### 3.3. Paromomycin Impacts on Host Survival

**Figure 3:**
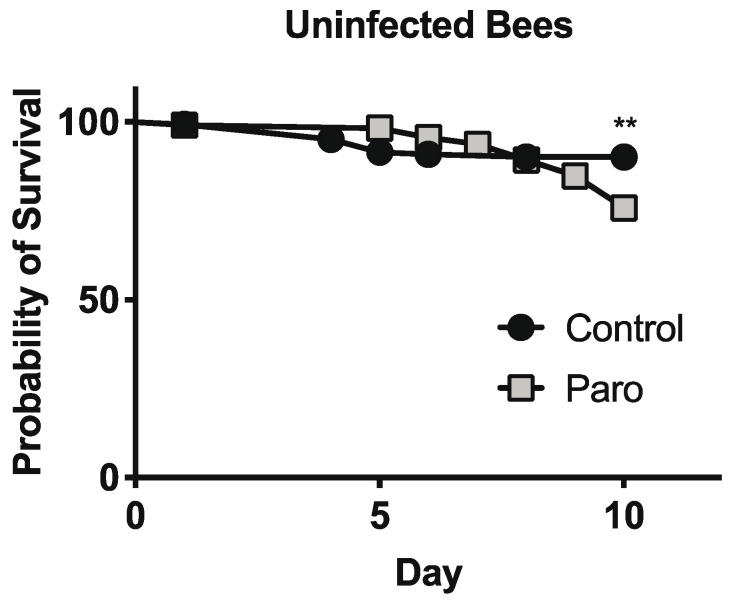
**Paromomycin impacts honey bee survival**. Survival of individual uninfected newly emerged bees fed with a sucrose solution alone (*n* = 142) or containing 1 mg/mL of paromomycin (*n* = 112) for 10 days. Statistical significance is noted as ** for *p* < 0.01.

To explore the effects of paromomycin on the host cells, we first examined honey bee survival during our experiments with infected bees. We found very low mortality among the bees after 4 days and no differences in the survival rates of infected bees that were fed paromomycin at 1 mg/mL for 4 days, starting 6 days post-eclosion (C = 96.4% survival, *n* = 192; paromomycin = 97.6% survival, *n* = 207; chi-squared test = 0.5156; df = 1; *p* = 0.4727). To better assess the impacts of paromomycin treatment on honey bee survival, newly emerged bees were fed a sucrose solution alone or containing paromomycin at 1 mg/mL for 10 days, starting 4 days post-eclosion. Here, we observed a decreased survival rate for uninfected bees that were fed paromomycin at 1 mg/mL (C = 90.1% survival, *n* = 142; paromomycin = 75.9% survival, *n* = 112; chi-squared test = 7.987; df = 1; *p* = 0.0047) (Figure 3).

### 3.4. Paromomycin Impacts on the Expression of General Stress Biomarker Genes and Specific Genes for Translation Inhibition

**Figure 4:**
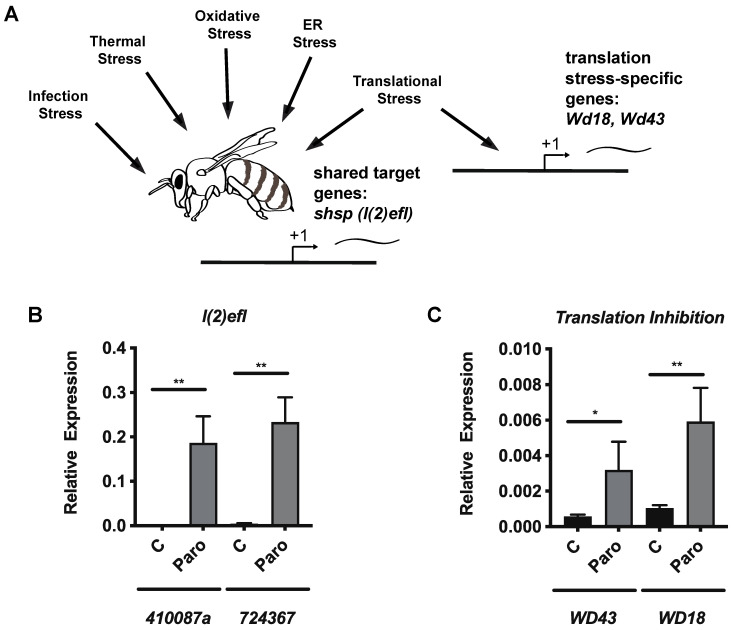
**Paromomycin impacts stress biomarker gene expression at doses that are effective at reducing *V. ceranae* infection intensity**. Schematic showing diverse stressors that induce the *l(2)efl* family of *shsp* genes and the translational stress-specific ribosome biogenesis genes *WD43* and *WD18* (**A**). Transcript levels of the *l(2)efl* genes *724367* and *410087a* (**B**) and the ribosome biogenesis genes *WD43* and *WD18* (**C**) relative to the *β-actin* in midgut tissue from adult bees captured at the landing board and fed with a sucrose solution alone (**C**, *n* = 10) or with 1 mg/mL of paromomycin (Paro, *n* = 10). Means ± SEM are shown and represent the expression values of the genes of interest, calculated using the 2^(−^^ΔCT)^ method for individual bees. Statistical significance is noted as * for *p* < 0.05 and ** for *p* < 0.01.

In light of the reduced survival of honey bees after paromomycin treatment, we wished to examine whether sublethal effects of paromomycin were observed in the honey bee cells. We measured the expression of selected *shsp* genes of the *l(2)efl* family, which have been identified as useful stress biomarker genes in honey bees [25,26,27] that respond to diverse types of stress (Figure 4A). Using qPCR, we found that the expression of *724367* and *410087a* increased in the midguts of uninfected bees that were treated with paromomycin relative to the control bees after 4 days of feeding (Figure 4B). To examine the expression of two genes that specifically mark translational stress in bees, we measured the expression of *WD repeat-containing protein 18* (*Wd18*) and *WD repeat-containing protein 43* (*Wd43*). We have previously shown that these genes are transcriptionally upregulated after translation inhibition by either halofuginone or cycloheximide, but not other forms of stress (Figure 2A) [28]. Using qPCR, we found that the expression of *WD43* and *WD18* increased in the midguts of uninfected bees that were treated with paromomycin relative to the control bees after 4 days of feeding (Figure 4C).

## 4. Discussion

Prior studies have found variable effects of paromomycin on microsporidia. One study showed a small effect of paromomycin on *V. apis* infection in honey bees [13]. However, cell culture-based studies have shown no effect of paromomycin on *E. cuniculi* [14] or *V. ceranae* [16] and a study of patients with *E. bieneusi* infection showed no change in infection level after paromomycin treatment [15]. The previous finding that paromomycin does not impact *V. ceranae* infection in cell culture models [16] is particularly noteworthy. In this study, the authors employed an innovative approach to test candidate anti-microsporidia agents by building on their previous development of a lepidopteran cell line that can be infected with *V. ceranae* spores and complete the entire intracellular life cycle while not producing infective spores [17]. While the specific reasons for the discordant observations of our two groups are unknown, they can likely be attributed to the differences between the models utilized. The infection system that we used is significantly more complex than the cell culture model due to the presence of the microbiome and whole organism physiology. Thus, the data from Gisder and Genersch may therefore provide support for the idea that paromomycin acts on *V. ceranae* indirectly in our system (see below). The cell culture study was also performed using a lepidopteran cell line in which only a single round of infection is achievable [17], potentially reducing its applicability to infection in caged honey bees. Despite the potential advantages of our system, it is important to note that experiments using caged bees cannot fully model the impact of a drug on *V. ceranae* infection or honey bee health in natural colony settings.

Microsporidia, as with fungi and other fungi-like organisms, have been shown to lack an essential binding site on the ribosome that is known to allow for maximal paromomycin inhibition in bacteria [12]. Therefore, it is interesting to speculate how these antibiotics may impact *V. ceranae* levels despite the latter lacking the canonical binding site. One aspect of microsporidia cell biology that may impact paromomycin resistance is the genome compaction that is observed in these organisms, which affects proteins that are involved in many cellular processes. Paromomycin binds to the small subunit of the eukaryotic ribosome, leading to a decrease in translation fidelity [29,30,31]. Mutations affecting many translation-related factors, including N-acetyltransferases [32], MetAP1 [33], and ribosome-associated chaperones (Ssb or RAC) [34], further sensitize cells to the proofreading inhibitory activity of paromomycin. *V. ceranae* apparently lacks these factors, which may impact its sensitivity to this drug. In fact, microsporidia appear to have a high basal error rate in protein synthesis, with a high degree of amino acid substitution [35]. This is likely due, in part, to changes in the aminoacyl-tRNA synthetase structure [35], but may also be compounded by the fact that the microsporidia ribosome apparently lacks the major rRNA expansion segments [36,37]. In particular, attachment sites for ribosomal proteins, such as eS31 and eL27, have been lost and the compacted ribosome found in microsporidia is hypothesized to have reduced quality control function [38,39]. It is interesting to speculate that paromomycin may have unexpected effects on microsporidia due to further eroding translational fidelity.

There is also a number of ways that this class of antibiotics may attenuate parasite levels through indirect effects operating outside of the microsporidia cell. First, paromomycin could alter the bacteria composition of the gut microbiome, such that *V. ceranae* growth is inhibited. Bacterial ribosomes are estimated to be 100- to 1000-fold more sensitive to aminoglycosides compared to most eukaryotic ribosomes [29] (with the exception of some protists, such as Leishmania [40]). Our results also indicated that paromomycin has impacts on the honey bee microbiome at much lower doses than those required to impact *V. ceranae* levels, suggesting that effects on the microbiome are not responsible for the reduction in *V. ceranae* infection intensity. In agreement, another study that used penicillin–streptomycin in bees found that such treatment increased *V. ceranae* infection levels [41]. However, it is known that the gut microbiome plays key roles in honey bee biology, making any treatment that causes deleterious effects on their microbiome potentially problematic [42]. Recent studies have shown that the gut microbiota of honey bees is more complex than that found in solitary insects [43] and that its composition can have a significant impact on honey bee health [44]. The microbiome community provides benefits to the honey bee host, including metabolic contributions [45] and immune modulation [46]. The perturbation of the honey bee microbiota by diverse mechanisms, such as antibiotic exposure or dietary alterations, can negatively impact honey bee health. Changes in the microbiome can impact the severity and outcome of infections by pathogenic microbes. Tetracycline exposure can lead to the outgrowth of opportunistic infections in the gut [47]. As described above, penicillin–streptomycin exposure can actually render bees more susceptible to *V. ceranae* infection instead of reducing infection levels [41], again arguing against the role of microbiome depletion in the *V. ceranae* reductions we observed. Microbiome composition is correlated with the infection intensity of *V. ceranae* infections in *A. mellifera* [48,49] and *A. cerana* [50] and can alter infection by another gut pathogen, *Lotmaria passim* [51]. Alterations in non-bacterial gut residents, such as yeasts [52,53], may also impact microsporidia infections. Paromomycin is known to impact the gut microbiota in humans after treatment [54] and our results show a pronounced reduction in all bacterial cells in the honey bee midgut after paromomycin treatment.

A second mechanism through which paromomycin could indirectly impact *V. ceranae* growth or survival is its effects on translation in the honey bee host cells. In fact, the analysis of the ribosomal RNA of *V. ceranae* [55] and *Apis mellifera* [56] leads to a prediction of paromomycin preferentially binding to the host rather than to the microsporidia ribosome (Moran Shalev-Benami, personal communication). There is a number of general mechanisms through which this could occur, including the following. First, paromomycin-treated cells may be functionally disrupted to a degree that prevents them from providing one or more resources that are key to microsporidia growth, such as ATP. Second, paromomycin-treated cells could be so damaged that they are dying before the parasite life cycle is completed. The sloughing of midgut epithelial cells can be observed in honey bees after exposure to other stressors [57]. Third, translation inhibition in the host cells may preferentially reduce the amount of one or more specific host proteins that are necessary for optimal parasite function. Finally, a translation inhibition-induced stress response that triggers immune pathways may be detrimental to microsporidia growth [58]. In the nematode, translational blockages induced by infection (which can be mimicked by the ribosome elongation inhibitor cycloheximide) can induce an immune response [59,60,61]. Conversely, in the fruit fly, pathogen-induced host translational blockages have been found to decrease both immune responses and epithelial renewal in the digestive tract [62]. We previously characterized the transcription response to translation inhibition in honey bee midgut tissue and identified a number of genes that are induced by translational stress (but not other types of stress), including *Wd18* and *Wd43* [28]. We found that both of these genes are induced after paromomycin treatment, suggesting that translation inhibition does occur. Although the cellular consequences of translation disruption in honey bees are not completely understood [18,28], a decrease in honey bee survival after paromomycin treatment is consistent with detrimental effects to the cell and tissue function. The increased expression of *shsp* genes also indicates that paromomycin has sublethal effects on honey bee midgut cells. The quantification of cellular stress responses via the measuring of HSP has been used as a surrogate to identify general organismal stress in a variety of settings, including the honey bee (reviewed in [63]). Based on the robust induction of some of the *l(2)efl* genes in response to a broad array of stressors, the quantification of these sHSP genes has been proposed as an optimal biomarker for honey bee stress [25,26,27], although the levels of these genes have not been directly linked to individual bee or colony-level health, as has been achieved for other biomarkers [64]. Some of the sHSP genes discussed here have been shown to be a part of the antiviral response in honey bees and bumble bees [65,66], suggesting that they may possess anti-*Vairimorpha* properties.

Our study only reported studies using caged bees. Thus, any efforts to pursue paromomycin for use by beekeepers would require rigorous field trials to assess the long-term effects of such a treatment strategy on honey bee health at the individual and colony levels. Critically, the doses of paromomycin that were necessary to reduce *V. ceranae* infection in our experiments have high activity against the bacteria species in the honey bee microbiome and can cause host cell stress and bee mortality. Thus, such a treatment strategy could cause widespread issues in agricultural settings. The authors of a previous study using an in vitro model of *V. ceranae* infection to identify anti-*Vairimorpha* agents also rightly pointed out that no antibiotics, such as paromomycin, would be an appropriate anti-*Vairimorpha* agent in the field due to the strictures on using antibiotics in honey bee colonies in many countries [16]. Thus, while paromomycin may prove to have anti-*Vairimorpha* activity in field studies, its value as a potential therapeutic is greatly diminished by it being a powerful antibiotic with activity against prokaryotes in the microbiome and having clear toxicity to honey bees.

## Supplementary Materials

The following supporting information can be downloaded at: https://www.mdpi.com/article/10.3390/microorganisms10061107/s1, Figure S1: Paromomycin treatment reduces V. ceranae infection levels in colony bees, Figure S2: Paromomycin alters the relative abundance of bacterial microbiome community members.

## References

[1] Weiss L.M. (2014). Microsporidia: Pathogens of Opportunity.

[2] Tokarev Y.S., Huang W.-F., Solter L.F., Malysh J.M., Becnel J.J., Vossbrinck C.R. (2020). A Formal Redefinition of the Genera *Nosema* and *Vairimorpha* (Microsporidia: Nosematidae) and Reassignment of Species Based on Molecular Phylogenetics. J. Invertebr. Pathol..

[3] Goblirsch M. (2017). *Nosema ceranae* Disease of the Honey Bee (*Apis mellifera*). Ann. Abeille..

[4] Martín-Hernández R., Bartolomé C., Chejanovsky N., Conte Y.L., Dalmon A., Dussaubat C., García-Palencia P., Meana A., Pinto M.A., Soroker V. (2018). *Nosema ceranae* in *Apis mellifera*: A 12 Years Postdetection Perspective. Environ. Microbiol..

[5] Snow J.W., Weiss L.M., Reinke A.W. (2022). Nosema apis and *N. ceranae* Infection in Honey Bees: A Model for Host-Pathogen Interactions in Insects. Microsporidia, Current Advances in Biology.

[6] Solter L.F., Becnel J.J., Oi D.H. (2012). Insect Pathology.

[7] Van den Heever J.P., Thompson T.S., Curtis J.M., Ibrahim A., Pernal S.F. (2014). Fumagillin: An Overview of Recent Scientific Advances and Their Significance for Apiculture. J. Agric. Food Chem..

[8] Mendoza Y., Diaz-Cetti S., Ramallo G., Santos E., Porrini M., Invernizzi C. (2017). *Nosema ceranae* Winter Control: Study of the Effectiveness of Different Fumagillin Treatments and Consequences on the Strength of Honey Bee (Hymenoptera: Apidae) Colonies. J. Econ. Entomol..

[9] Huang W.-F., Solter L.F., Yau P.M., Imai B.S. (2013). *Nosema ceranae* Escapes Fumagillin Control in Honey Bees. PLoS Pathog..

[10] Huntsman E.M., Cho R.M., Kogan H.V., McNamara-Bordewick N.K., Tomko R.J., Snow J.W. (2021). Proteasome Inhibition Is an Effective Treatment Strategy for Microsporidia Infection in Honey Bees. Biomolecules.

[11] Krause K.M., Serio A.W., Kane T.R., Connolly L.E. (2016). Aminoglycosides: An Overview. Cold Spring Harb. Perspect. Med..

[12] Katiyar S.K., Visvesvara G.S., Edlind T.D. (1995). Comparisons of Ribosomal RNA Sequences from Amitochondrial Protozoa: Implications for Processing, MRNA Binding and Paromomycin Susceptibility. Gene.

[13] Moffett J.O., Lackett J.J., Hitchcock J.D. (1969). Compounds Tested for Control of Nosema in Honey Bees. J. Econ. Entomol..

[14] Beauvais B., Sarfati C., Challier S., Derouin F. (1994). In Vitro Model to Assess Effect of Antimicrobial Agents on *Encephalitozoon cuniculi*. Antimicrob. Agents Chemother..

[15] Van Gool T., Snijders F., Reiss P., Schattenkerk J.K.E., van den Bergh Weerman M.A., Bartelsman J.F., Bruins J.J., Canning E.U., Dankert J. (1993). Diagnosis of Intestinal and Disseminated Microsporidial Infections in Patients with HIV by a New Rapid Fluorescence Technique. J. Clin. Pathol..

[16] Gisder S., Genersch E. (2015). Identification of Candidate Agents Active against *N. ceranae* Infection in Honey Bees: Establishment of a Medium Throughput Screening Assay Based on *N. ceranae* Infected Cultured Cells. PLoS ONE.

[17] Gisder S., Moeckel N., Linde A., Genersch E. (2011). A Cell Culture Model for *Nosema ceranae* and *Nosema apis* Allows New Insights into the Life Cycle of These Important Honey Bee-Pathogenic Microsporidia. Environ. Microbiol..

[18] Snow J.W. (2020). Prolyl-tRNA Synthetase Inhibition Reduces Microsporidia Infection Intensity in Honey Bees. Apidologie.

[19] Holt H.L., Aronstein K.A., Grozinger C.M. (2013). Chronic Parasitization by *Nosema* Microsporidia Causes Global Expression Changes in Core Nutritional, Metabolic and Behavioral Pathways in Honey Bee Workers (*Apis mellifera*). BMC Genom..

[20] Fries I., Chauzat M.-P., Chen Y.P., Doublet V., Genersch E., Gisder S., Higes M., McMahon D.P., Martín-Hernández R., Natsopoulou M. (2013). Standard Methods for *Nosema* Research. J. Apic. Res..

[21] Cantwell G.E. (1970). Standard Methods for Counting *Nosema* Spores. Am. Bee J..

[22] Snow J.W., Koydemir H.C., Karinca D.K., Liang K., Tseng D., Ozcan A. (2019). Rapid Imaging, Detection, and Quantification of *Nosema ceranae* spores in Honey Bees Using Mobile Phone-Based Fluorescence Microscopy. Lab A Chip.

[23] Schmittgen T.D., Livak K.J. (2008). Analyzing Real-Time PCR Data by the Comparative C(T) Method. Nat. Protoc..

[24] Kešnerová L., Mars R.A.T., Ellegaard K.M., Troilo M., Sauer U., Engel P. (2017). Disentangling Metabolic Functions of Bacteria in the Honey Bee Gut. PLoS Biol..

[25] Shih S.R., Bach D.M., Rondeau N.C., Sam J., Lovinger N.L., Lopatkin A.J., Snow J.W. (2021). Honey Bee SHSP Are Responsive to Diverse Proteostatic Stresses and Potentially Promising Biomarkers of Honey Bee Stress. Sci. Rep.

[26] McAfee A., Milone J., Chapman A., Foster L.J., Pettis J.S., Tarpy D.R. (2020). Candidate Stress Biomarkers for Queen Failure Diagnostics. BMC Genom..

[27] McAfee A., Chapman A., Higo H., Underwood R., Milone J., Foster L.J., Guarna M.M., Tarpy D.R., Pettis J.S. (2020). Vulnerability of Honey Bee Queens to Heat-Induced Loss of Fertility. Nat. Sustain..

[28] Flores M.E., McNamara-Bordewick N.K., Lovinger N.L., Snow J.W. (2021). Halofuginone Triggers a Transcriptional Program Centered on Ribosome Biogenesis and Function in Honey Bees. Insect Biochem. Molec..

[29] Fan-Minogue H., Bedwell D.M. (2008). Eukaryotic Ribosomal RNA Determinants of Aminoglycoside Resistance and Their Role in Translational Fidelity. RNA.

[30] De Loubresse N.G., Prokhorova I., Holtkamp W., Rodnina M.V., Yusupova G., Yusupov M. (2014). Structural Basis for the Inhibition of the Eukaryotic Ribosome. Nature.

[31] Prokhorova I., Altman R.B., Djumagulov M., Shrestha J.P., Urzhumtsev A., Ferguson A., Chang C.-W.T., Yusupov M., Blanchard S.C., Yusupova G. (2017). Aminoglycoside Interactions and Impacts on the Eukaryotic Ribosome. Proc. Natl. Acad. Sci. USA.

[32] Kamita M., Kimura Y., Ino Y., Kamp R.M., Polevoda B., Sherman F., Hirano H. (2011). Nα-Acetylation of Yeast Ribosomal Proteins and Its Effect on Protein Synthesis. J. Proteom..

[33] Fujii K., Susanto T.T., Saurabh S., Barna M. (2018). Decoding the Function of Expansion Segments in Ribosomes. Mol. Cell.

[34] Rakwalska M., Rospert S. (2004). The Ribosome-Bound Chaperones RAC and Ssb1/2p Are Required for Accurate Translation in Saccharomyces Cerevisiae. Mol. Cell. Biol..

[35] Melnikov S.V., Rivera K.D., Ostapenko D., Makarenko A., Sanscrainte N.D., Becnel J.J., Solomon M.J., Texier C., Pappin D.J., Söll D. (2018). Error-Prone Protein Synthesis in Parasites with the Smallest Eukaryotic Genome. Proc. Natl. Acad. Sci. USA.

[36] Peyretaillade E., Biderre C., Peyret P., Duffieux F., Méténier G., Gouy M., Michot B., Vivarès C.P. (1998). Microsporidian *Encephalitozoon cuniculi,* a Unicellular Eukaryote with an Unusual Chromosomal Dispersion of Ribosomal Genes and a LSU RRNA Reduced to the Universal Core. Nucleic Acids Res..

[37] Vossbrinck C.R., Maddox J.V., Friedman S., Debrunner-Vossbrinck B.A., Woese C.R. (1987). Ribosomal-Rna Sequence Suggests Microsporidia Are Extremely Ancient Eukaryotes. Nature.

[38] Melnikov S., Manakongtreecheep K., Rivera K., Makarenko A., Pappin D., Söll D. (2018). Muller’s Ratchet and Ribosome Degeneration in the Obligate Intracellular Parasites Microsporidia. Int. J. Mol. Sci..

[39] Barandun J., Hunziker M., Vossbrinck C.R., Klinge S. (2019). Evolutionary Compaction and Adaptation Visualized by the Structure of the Dormant Microsporidian Ribosome. Nat. Microbiol..

[40] Shalev-Benami M., Zhang Y., Rozenberg H., Nobe Y., Taoka M., Matzov D., Zimmerman E., Bashan A., Isobe T., Jaffe C.L. (2017). Atomic Resolution Snapshot of *Leishmania* Ribosome Inhibition by the Aminoglycoside Paromomycin. Nat. Commun..

[41] Li J.H., Evans J.D., Li W.F., Zhao Y.Z., DeGrandi-Hoffman G., Huang S.K., Li Z.G., Hamilton M., Chen Y.P. (2017). New Evidence Showing That the Destruction of Gut Bacteria by Antibiotic Treatment Could Increase the Honey Bee’s Vulnerability to *Nosema* Infection. PLoS ONE.

[42] Engel P., Moran N.A. (2013). The Gut Microbiota of Insects—Diversity in Structure and Function. FEMS Microbiol. Rev..

[43] Kwong W.K., Medina L.A., Koch H., Sing K.-W., Soh E.J.Y., Ascher J.S., Jaffé R., Moran N.A. (2017). Dynamic Microbiome Evolution in Social Bees. Sci. Adv..

[44] Raymann K., Moran N.A. (2018). The Role of the Gut Microbiome in Health and Disease of Adult Honey Bee Workers. Curr. Opin. Insect Sci..

[45] Zheng H., Powell J.E., Steele M.I., Dietrich C., Moran N.A. (2017). Honeybee Gut Microbiota Promotes Host Weight Gain via Bacterial Metabolism and Hormonal Signaling. Proc. Natl. Acad. Sci. USA.

[46] Kwong W.K., Mancenido A.L., Moran N.A. (2017). Immune System Stimulation by the Native Gut Microbiota of Honey Bees. R. Soc. Open Sci..

[47] Raymann K., Shaffer Z., Moran N.A. (2017). Antibiotic Exposure Perturbs the Gut Microbiota and Elevates Mortality in Honeybees. PLoS Biol..

[48] Maes P.W., Rodrigues P.A.P., Oliver R., Mott B.M., Anderson K.E. (2016). Diet Related Gut Bacterial Dysbiosis Correlates with Impaired Development, Increased Mortality and Nosema Disease in the Honey Bee (*Apis mellifera*). Mol. Ecol..

[49] Rubanov A., Russell K.A., Rothman J.A., Nieh J.C., Mcfrederick Q.S. (2019). Intensity of *Nosema ceranae* Infection Is Associated with Specific Honey Bee Gut Bacteria and Weakly Associated with Gut Microbiome Structure. Sci. Rep..

[50] Huang S.K., Ye K.T., Huang W.F., Ying B.H., Su X., Lin L.H., Li J.H., Chen Y.P., Li J.L., Bao X.L. (2018). Influence of Feeding Type and *Nosema ceranae* Infection on the Gut Microbiota of *Apis cerana* Workers. mSystems.

[51] Schwarz R.S., Moran N.A., Evans J.D. (2016). Early Gut Colonizers Shape Parasite Susceptibility and Microbiota Composition in Honey Bee Workers. Proc. Natl. Acad. Sci. USA.

[52] Ptaszyńska A.A., Paleolog J., Borsuk G. (2016). *Nosema ceranae* Infection Promotes Proliferation of Yeasts in Honey Bee Intestines. PLoS ONE.

[53] Tauber J.P., Nguyen V., Lopez D., Evans J.D. (2019). Effects of a Resident Yeast from the Honeybee Gut on Immunity, Microbiota, and Nosema Disease. Insects.

[54] Heinsen F.-A., Knecht H., Neulinger S.C., Schmitz R.A., Knecht C., Kühbacher T., Rosenstiel P.C., Schreiber S., Friedrichs A.K., Ott S.J. (2015). Dynamic Changes of the Luminal and Mucosa-Associated Gut Microbiota during and after Antibiotic Therapy with Paromomycin. Gut Microbes.

[55] Sagastume S., Martín-Hernández R., Higes M., Henriques-Gil N. (2016). Genotype Diversity in the Honey Bee Parasite *Nosema ceranae:* Multi-Strain Isolates, Cryptic Sex or Both?. BMC Evol. Biol..

[56] Gillespie J.J., Johnston J.S., Cannone J.J., Gutell R.R. (2006). Characteristics of the Nuclear (18S, 5.8S, 28S and 5S) and Mitochondrial (12S and 16S) RRNA Genes of *Apis mellifera* (Insecta: Hymenoptera): Structure, Organization, and Retrotransposable Elements. Insect Mol. Biol..

[57] Bach D.M., Holzman M.A., Wague F., Miranda J., Lopatkin A.J., Mansfield J.H., Snow J.W. (2021). Thermal Stress Induces Tissue Damage and a Broad Shift in Regenerative Signaling Pathways in the Honey Bee Digestive Tract. J. Exp. Biol..

[58] Fischer N.L., Naseer N., Shin S., Brodsky I.E. (2020). Effector-Triggered Immunity and Pathogen Sensing in Metazoans. Nat. Microbiol..

[59] Melo J.A., Ruvkun G. (2012). Inactivation of Conserved *C. elegans* Genes Engages Pathogen- and Xenobiotic-Associated Defenses. Cell.

[60] Dunbar T.L., Yan Z., Balla K.M., Smelkinson M.G., Troemel E.R. (2012). *C. elegans* Detects Pathogen-Induced Translational Inhibition to Activate Immune Signaling. Cell Host Microbe.

[61] McEwan D.L., Kirienko N.V., Ausubel F.M. (2012). Host Translational Inhibition by *Pseudomonas aeruginosa* Exotoxin A Triggers an Immune Response in *Caenorhabditis elegans*. Cell Host Microbe.

[62] Chakrabarti S., Liehl P., Buchon N., Lemaitre B. (2012). Infection-Induced Host Translational Blockage Inhibits Immune Responses and Epithelial Renewal in the *Drosophila* Gut. Cell Host Microbe.

[63] Even N., Devaud J.-M., Barron A. (2012). General Stress Responses in the Honey Bee. Insects.

[64] López-Uribe M.M., Ricigliano V.A., Simone-Finstrom M. (2019). Defining Pollinator Health: Assessing Bee Ecological, Genetic, and Physiological Factors at the Individual, Colony, and Population Levels. Annu. Rev. Anim. Biosci..

[65] Brutscher L.M., Daughenbaugh K.F., Flenniken M.L. (2017). Virus and DsRNA-Triggered Transcriptional Responses Reveal Key Components of Honey Bee Antiviral Defense. Sci. Rep..

[66] McMenamin A., Daughenbaugh K., Parekh F., Pizzorno M., Flenniken M. (2018). Honey Bee and Bumble Bee Antiviral Defense. Viruses.

