# Supplementary material for: Paromomycin Reduces Vairimorpha (Nosema) ceranae Infection in Honey Bees but Perturbs Microbiome Levels and Midgut Cell Function
Source: Microorganisms. 2022 May 27;10(6):1107. doi: 10.3390/microorganisms10061107 (PMC9231153; doi:10.3390/microorganisms10061107)
Supplement: Supplementary file 1 [file microorganisms-10-01107-s001.zip › microorganisms-1708119-supplementary.pdf]

Supplemental Figure Legends

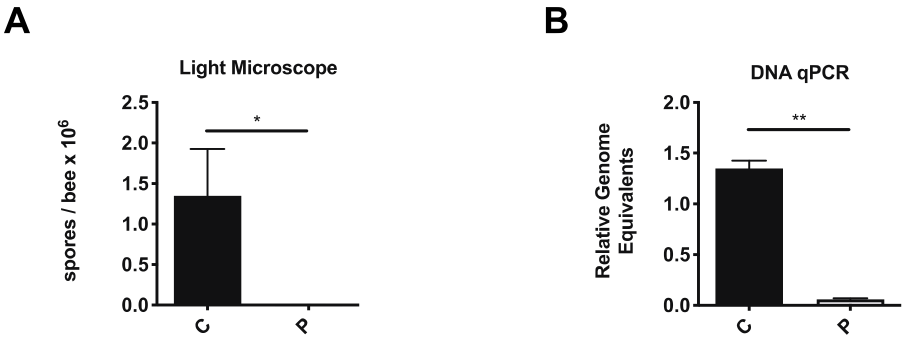

Figure S1. Paromomycin treatment reduces *V. ceranae* infection level in colony bees. *V. ceranae* levels in midguts of infected landing board bees fed sucrose syrup containing various doses of Paromomycin for 4 days as determined by spore count using light microscopy (A) or by qPCR (B). Statistical significance is noted as * for $p < 0.05$ and ** for $p < 0.01$.

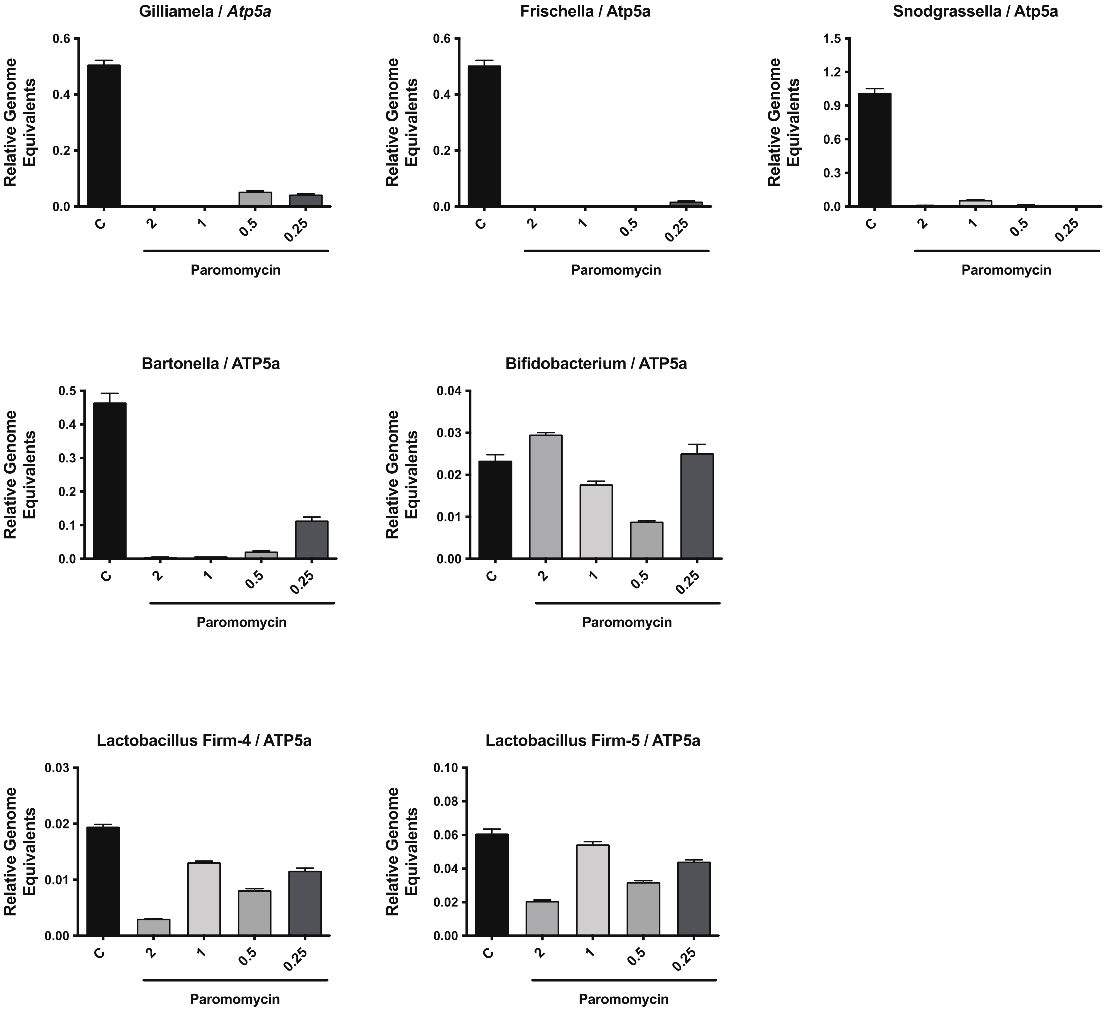

Figure S2. Paromomycin alters relative abundance of bacterial microbiome community members.

Levels of the major species of bacterial microbiome community members (*Gilliamella apicola, Frischella perrara, Snodgrassella alvi, Bartonella apis, Bifidobacterium asteroids, Lactobacillus* Firm-4, and *Lactobacillus* Firm-5) as determined by qPCR in midguts of infected newly eclosed bees fed sucrose syrup containing various doses of paromomycin for 4 days.
